# Impact of coronavirus disease 2019 on respiratory surveillance and explanation of high detection rate of human rhinovirus during the pandemic in the Republic of Korea

**DOI:** 10.1111/irv.12894

**Published:** 2021-08-18

**Authors:** Heui Man Kim, Eun Jung Lee, Nam‐Joo Lee, Sang hee Woo, Jeong‐Min Kim, Jee Eun Rhee, Eun‐Jin Kim

**Affiliations:** ^1^ Division of Emerging Infectious Diseases, Bureau of Infectious Disease Diagnosis Control Korea Disease Control and Prevention Agency Cheongju Republic of Korea; ^2^ Division of Laboratory Diagnosis Analysis, Regional Centers for Disease Control and Prevention Korea Disease Control and Prevention Agency Incheon Republic of Korea

**Keywords:** COVID‐19, respiratory virus, rhinovirus, surveillance

## Abstract

**Background:**

After the detection of the first case of coronavirus disease 2019 (COVID‐19) in South Korea on January 20, 2019, it has triggered three major outbreaks. To decrease the disease burden of COVID‐19, social distancing and active mask wearing were encouraged, reducing the number of patients with influenza‐like illness and altering the detection rate of influenza and respiratory viruses in the Korea Influenza and Respiratory Viruses Surveillance System (KINRESS). We examined the changes in respiratory viruses due to COVID‐19 in South Korea and virological causes of the high detection rate of human rhinovirus (hRV) in 2020.

**Methods:**

We collected 52 684 oropharyngeal or nasopharyngeal swab samples from patients with influenza‐like illness in cooperation with KINRESS from 2016 to 2020. Influenza virus and other respiratory viruses were confirmed using real‐time RT‐PCR. The weekly detection rate was used to compare virus detection patterns.

**Results:**

Non‐enveloped virus (hRV, human bocavirus, and human adenovirus) detection rates during the COVID‐19 pandemic were maintained. The detection rate of hRV significantly increased in 2020 compared with that in 2019 and was negatively correlated with number of COVID‐19‐confirmed cases in 2020. The distribution of strains and genetic characteristics in hRV did not differ between 2019 and 2020.

**Conclusions:**

The COVID‐19 pandemic impacted the respiratory virus detection rate. The extremely low detection rate of enveloped viruses resulted from efforts to prevent the spread of COVID‐19 in South Korea. The high detection rate of hRV may be related to resistance against environmental conditions as a non‐enveloped virus and the long period of viral shedding from patients.

## 1 INTRODUCTION

Coronavirus disease 2019 (COVID‐19) is an infectious disease caused by a newly discovered coronavirus that emerged in Wuhan City, Hubei province in China, on December 31, 2019.^1^ After COVID‐19 was first detected on January 2020 in South Korea, the first wave emerged around Deagu City and Gyungbuk province from February to March, the second wave emerged in the Metropolitan in August and September 2020,^2^, ^3^ and the third wave began in November 2020.^4^ The spread of COVID‐19 was slowed by implementation of high‐intensity social distancing and isolation measures as well as easily available testing for COVID‐19 in screening centers in all cities and provinces.^5^, ^6^ These active precautions led to reduction in the number of patients with influenza‐like illness (ILI) and lower numbers of respiratory specimens collected by the national surveillance program for respiratory virus (Korea Influenza and Respiratory Viruses Surveillance System, KINRESS).^7^ According to our national surveillance report, based on the weekly detection rates of influenza and respiratory viruses for 4 years (2016–2019) by KINRESS, influenza virus (IFV), human respiratory syncytial virus (hRSV), human parainfluenza virus (hPIV), human coronavirus (hCoV), human metapneumo virus (hMPV), and human bocavirus (hBoV) showed seasonal detection patterns. However, human adenovirus (hAdV) was maintained at a steady low detection rate annually and human rhinovirus (hRV) was highly detected in patients with ILI. The detection rate and patterns of respiratory viruses in KINRESS were altered in 2020. In this study, we evaluated the changes in national surveillance for respiratory viruses during the COVID‐19 pandemic in South Korea and the virological causes of these changes. This study was conducted for the first time in South Korea to analyze detection pattern of respiratory viruses that have changed significantly since COVID‐19 and to establish an effective quarantine policy for COVID‐19 and other viral respiratory diseases.

## 2 MATERIALS AND METHODS

### 2.1 Respiratory virus surveillance system

The Korea Disease Control and Prevention Agency has been performing national surveillance for KINRESS since 2000.^8^ The system includes 63 clinics and 18 regional laboratories (Public Health and Environment Research Institute, PHERI). The clinic provides up to eight upper respiratory specimens per week collected from patients with ILI, a measured temperature of ≥38°C, and cough, with an onset within the past 10 days.^9^ PHERI conducts real‐time reverse transcription‐polymerase chain reaction (PCR) or real‐time PCR to detect IFV, hRSV, hPIV, hCoV, hMPV, hBoV, hAdV, and hRV and reports the diagnosis results to the Korea Disease Control and Prevention Agency, who shares the national respiratory virus surveillance data with the public through the National Influenza Center (NIC).

### 2.2 Clinical specimen collection

Oropharyngeal or nasopharyngeal swab samples (52 684 samples) were collected from patients with ILI through KINRESS from 2016 to 2020. The specimens were delivered to PHERI for molecular diagnosis. The oropharyngeal or nasopharyngeal swabs were stored in Viral Transport Media (BD Biosciences, Franklin Lakes, NJ, USA) before analysis.

### 2.3 RNA or DNA extraction

Viral RNA or DNA was extracted from 140 μl of sample medium using the MagNA Pure 96 Instrument (Roche Life Science, Basel, Switzerland) according to the manufacturer's instructions (Magna Pure 96 DNA and RAN NA small volume kit).

### 2.4 Genetic analysis of influenza and other respiratory viruses

Five commercial respiratory virus detection real‐time one‐step reverse transcription‐PCR kits (Kogene Bio, Seoul, South Korea) were used to detect the viral nucleic acids of 15 subtypes of eight respiratory viruses, including hRSVs (type A, B), IFVs (type A/H1N1pdm09, A/H3N2, B), hPIVs (type 1, 2, 3), HCoVs (type OC43, 229E, NL63), hRV, hAdV, hBoV, and hMPV ^10^. Viral cDNAs, except those from hAdV and hBoV, were synthesized from 5 μl of extracted nucleic acids by reverse transcriptase at 50°C for 30 min, followed by inactivation of reverse transcriptase at 95°C for 10 min. PCR amplification was performed with 40 cycles of 95°C for 15 s and 60°C for 1 min in an ABI 7500 Fast instrument (Applied Biosystems, Foster City, CA, USA). Amplified PCR products were sequenced by Macrogen (Seoul, Korea). We used **CLC Workbench** v X. X (**CLC** bio, Aarhus, Denmark) for **sequencing** and assembly.

### 2.5 Statistical analysis

We used 52 684 oropharyngeal or nasopharyngeal swab samples, with the weekly detection rate calculated from 55 825 tested including co‐infection cases. The weekly detection rate of eight respiratory viruses (hRSV, IFV, hPIV, hCoV, hRV, hAdV, hBoV, and hMPV) from 2016 to 2020 in KINRESS and that of COVID‐19‐confirmed cases from 2020 was used for statistical analysis (supporting information S1). R software (ver. 4.0.2; The R Project for Statistical Computing, Vienna, Austria) was used for most analyses.^11^ The statistical significance of the means between two independent groups was analyzed by Welch's *t*‐test. A *P*‐value < 0.05 was considered to indicate statistically significant results. Pearson correlation coefficient (*R*) was calculated to determine the correlation between severe acute respiratory syndrome coronavirus 2 and hRV using the R heatmap function.

### 2.6 Sequencing and analysis

We randomly selected 94 samples from 2019 and 2020 for amplification using primers, generating a 635‐base pair fragment containing part of the 5′ untranslated region and all of VP4 and part of the VP2 regions.^12^, ^13^, ^14^ PCR amplicons were purified using the QIA PCR purification kit (Qiagen, Hilden, Germany) and sequenced with appropriate primers^12^ in both directions on an ABI‐3100 Prism Genetic Analyzer using the BigDye Terminator version 3.1 sequencing kit (Applied Biosystems). The sequences were compared with all available sequences in the GenBank database^15^ using BLASTN (http://blast.ncbI.nlm.nih.gov/Blast.cgi) tools to differentiate between the hRV A, B, and C species.

The nucleotide sequences were aligned using the MUSCLE program in MEGA7 (ver. 7.0.26).^16^ Phylogenetic analysis was performed with the 94 generated sequences and 19 reference sequences using RAxML (ver.8.1.21).^17^ Maximum likelihood trees with 1000 bootstraps were constructed based on the general time reversible + gamma distribution + proportion of invariable sites model after testing for the best‐fit model of nucleotide substitution in jModelTest (ver. 2.1.10).^18^

### 2.7 Submission to GenBank

Ninety‐four sequences were submitted to GenBank with part of the 5′ untranslated region and all of the VP4 and part of the VP2 regions. The sequences generated in this study were assigned GenBank accession numbers are given in supporting information S2.

### 2.8 Ethics statement

The study protocol was reviewed and approved by the institutional review board of the Korea Disease Control and Prevention Agency (Ethics number: 2016‐05‐02‐C‐A).

## 3 RESULTS

### 3.1 Comparison of influenza and respiratory virus weekly detection rate between 2020 and 2016–2019

**FIGURE 1:**
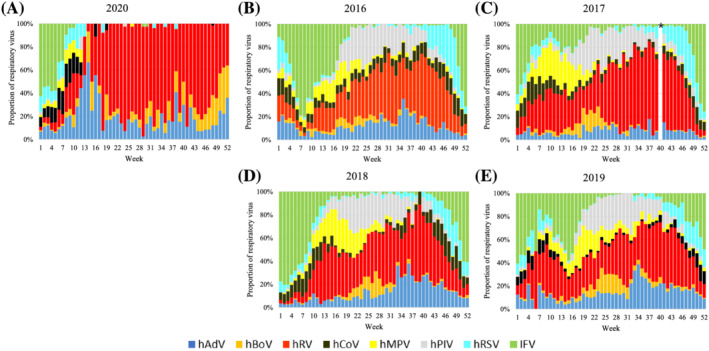
Proportion of respiratory viruses detected in patients with influenza‐like illness each year. (A) 2020, (B) 2016, (C) 2017, (D) 2018, and (E) 2019. *No data because of national holiday

**FIGURE 2:**
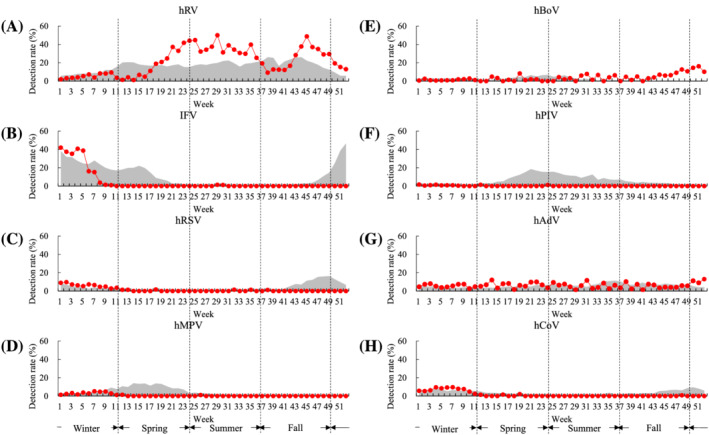
Comparison of weekly detection rate of eight respiratory viruses in 2020 and recent 4 years (2016–2019) in Korea Influenza and Respiratory Viruses Surveillance System (KINRESS). The gray background indicates weekly detection rate in 2016–2019, and red line indicates weekly detection rate in 2020. (A) Human rhinovirus (hRV), (B) influenza virus (IFV), (C) human respiratory syncytial virus, (D) human metapneumovirus (hMPV), (E) human bocavirus, (F) human parainfluenza virus, (G) human adenovirus (hAdV), and (H) human corona virus

KINRESS has surveyed eight respiratory viruses (IFV, hRV, hRSV, hMPV, hBoV, hPIV, hAdV, and hCoV) in patients with ILI visiting clinical centers, South Korea. Unusual changes have been observed in the proportion of detected respiratory viruses since the outbreak COVID‐19 (Figure 1). Most viruses showed seasonal trends in prevalence over the last 4 years (2016–2019), as shown in Figure 2. hRV was most frequently detected infection in patients with ILI during all weeks of the year. Particularly, the hRV detection rate decreased significantly during the influenza epidemic season (winter). IFV was commonly detected in winter and spring and rarely detected in summer. hRSV was accompanied by IFV in winter and fall and showed the highest detection rate late in the year just before the influenza epidemic. The detection rate of hMPV was increased immediately after the influenza epidemic early in the year, and hBoV and hPIV (1, 2, and 3) showed high detection rates in spring and summer. Although the detection rate hCoV (229E, NL63, and OC43) was elevated during the influenza epidemic season, the hAdV was detected under 10% during all seasons but slightly increased in late summer.

**FIGURE 3:**
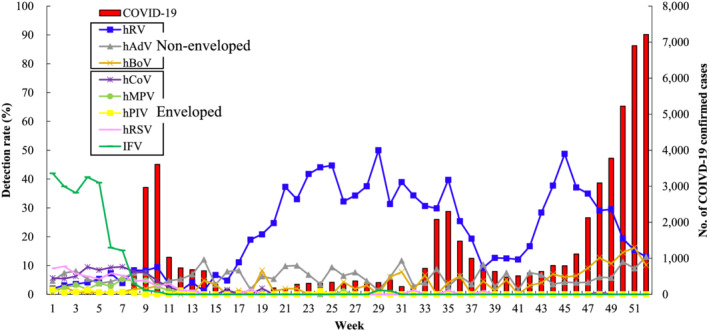
Weekly detection rate of eight respiratory viruses and SARS‐CoV2 in 2020. The bar filled with red indicates the number of confirmed COVID‐19 cases in 2020, South Korea. Blue, gray, and orange lines indicate the non‐enveloped human rhinovirus (hRV), human adenovirus (hAdV), and human bocavirus (hBoV), respectively. Purple, light green, yellow, pink, and deep green lines indicate the enveloped human coronavirus (hCoV), human metapneumovirus (hMPV), human parainfluenza virus (hPIV), human respiratory syncytial virus (hRSV), and influenza virus (IFV), respectively

**FIGURE 4:**
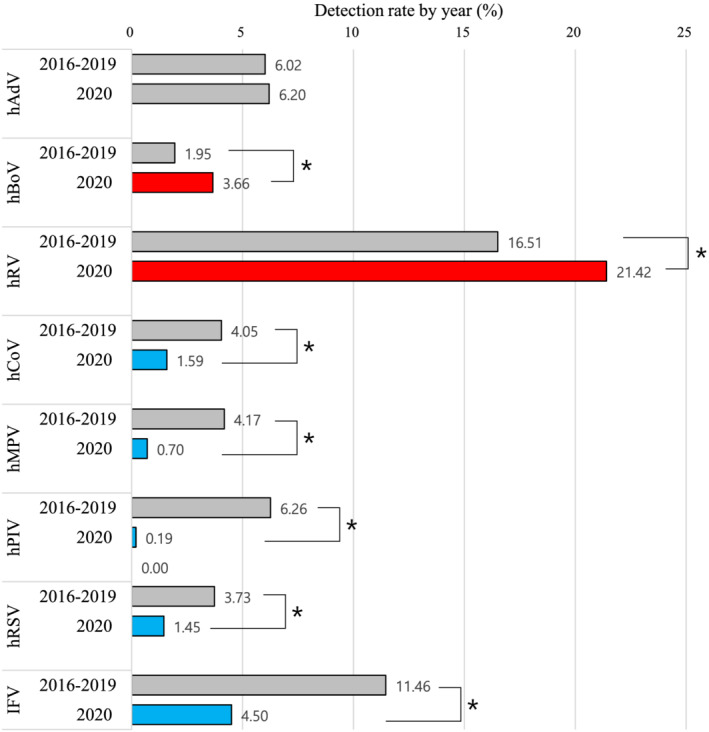
Comparison of detection rate of eight respiratory viruses in 2020 and recent 4 years (2016–2019). The bars filled with red or blue indicate significant increases or decreases, respectively (*, *P* < 0.05)

The COVID‐19 epidemic affected the detection rate of influenza and other respiratory viruses. After the first wave of COVID‐19, although enveloped viruses (IFV, hCoV, hMPV, hPIV, and hRSV) were not detected, non‐enveloped viruses (hRV, hBoV, and hAdV) continued to be detected in patients with ILI (Figure 3). Even the most distinctly seasonal IFV was has not been detected since the start of the COVID‐19 pandemic in 2020. Comparison of the weekly detection rates of influenza and seven respiratory viruses between 2016–2019 and 2020 showed that the weekly detection rates of IFV, hCoV, hMPV, hPIV, and hRSV were significantly decreased, whereas those of hRV and hBoV were significantly increased in 2020 (Figure 4).

Over 4 years (2016–2019), IFV was detected in patients with ILI at an annual rate of 11.46%, with IFV type A dominant in early winter and followed by IFV type B in late winter and spring. The annual detection rate of hRV was 16.51%, which was the highest among the respiratory viruses evaluated. hRSV is annually detected in 3.73% of patients with ILI and is typically restricted to infants and toddlers in fall and early winter. hADV was detected at a rate of 6.02% in patients with ILI and showed no distinct seasonality. However, some sporadic hADV cases were reported in small outbreak from swimming pool during summer. hPIV type 1, 2, and 3 showed an annual detection rate of 6.26% and was prevalent in summer. hBoV showed the lowest annual detection rate (1.95%) and was coinfected with other respiratory viruses. hMPV was detected mainly in spring with an annual detection rate of 4.17%. Common hCoVs including NL63, OC43, and 229E circulated during influenza season with an annual detection rate of 4.05%.

### 3.2 Coefficient of correlation between COVID‐19 and respiratory viruses

**FIGURE 5:**
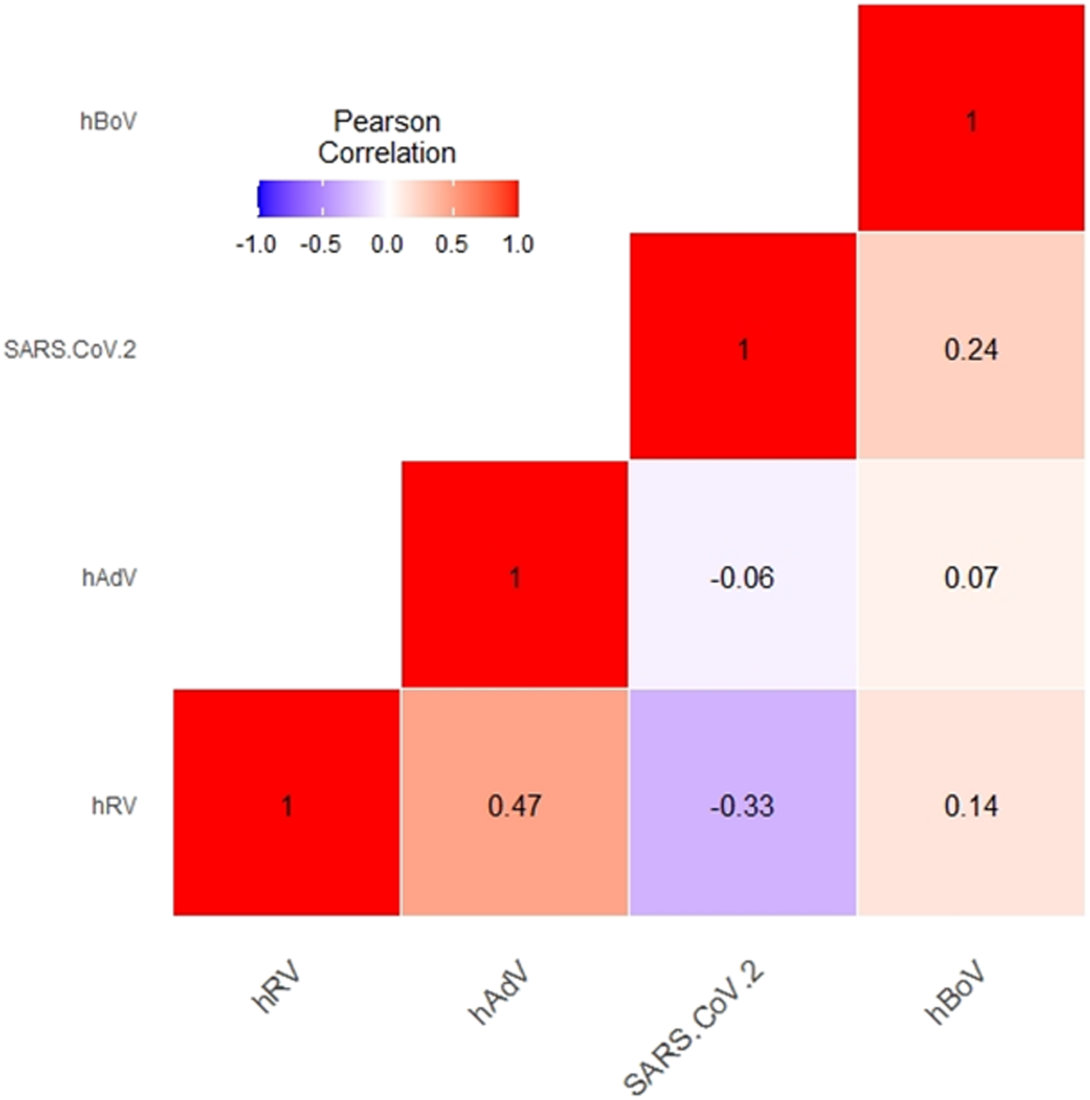
Pearson correlation coefficient (*R*) was calculated to estimate negative or positive interaction between SARS‐CoV2 and non‐enveloped viruses (human rhinovirus [hRV], human adenovirus [hAdV], and human bocavirus [hBoV]) using R heatmap function. Negative correlations are in blue and positive correlations are in red. *P*‐value for comparison of SARS‐CoV2 and hRV was 0.0202

There have been three waves of the COVID‐19 pandemic in South Korea. The first wave occurred in Deagu City and Gyungbuk province at around Week 10, the second wave occurred in Metropolitan at around Week 35, and the third wave occurred nationwide at Week 52 in 2020. Only non‐enveloped viruses (hRV, hBoV, and hAdV) were detected during the COVID‐19 waves. Although hAdV and hBoV were detected at rates of around 10% in patients with ILI, hRV was detected in up to 50% of these patients. hRV showed two peaks of infection, both of which occurred when the number of COVID‐19 cases was below 1000 cases in 1 week (Figure 3A). The Pearson correlation coefficient was calculated between COVID‐19 and non‐enveloped viruses (hRV, hBoV, and hAdV) that were maintained during the COVID‐19 pandemic. Although the weekly detection rate of hBoV or hAdV was not significantly related to the COVID‐19 pandemic pattern, hRV was negatively correlated with COVID‐19 (correlation coefficient value −0.33, *P* = 0.02) (Figure 5).

### 3.3 Comparison of hRV detection rate according to age between 2019 and 2020

**FIGURE 6:**
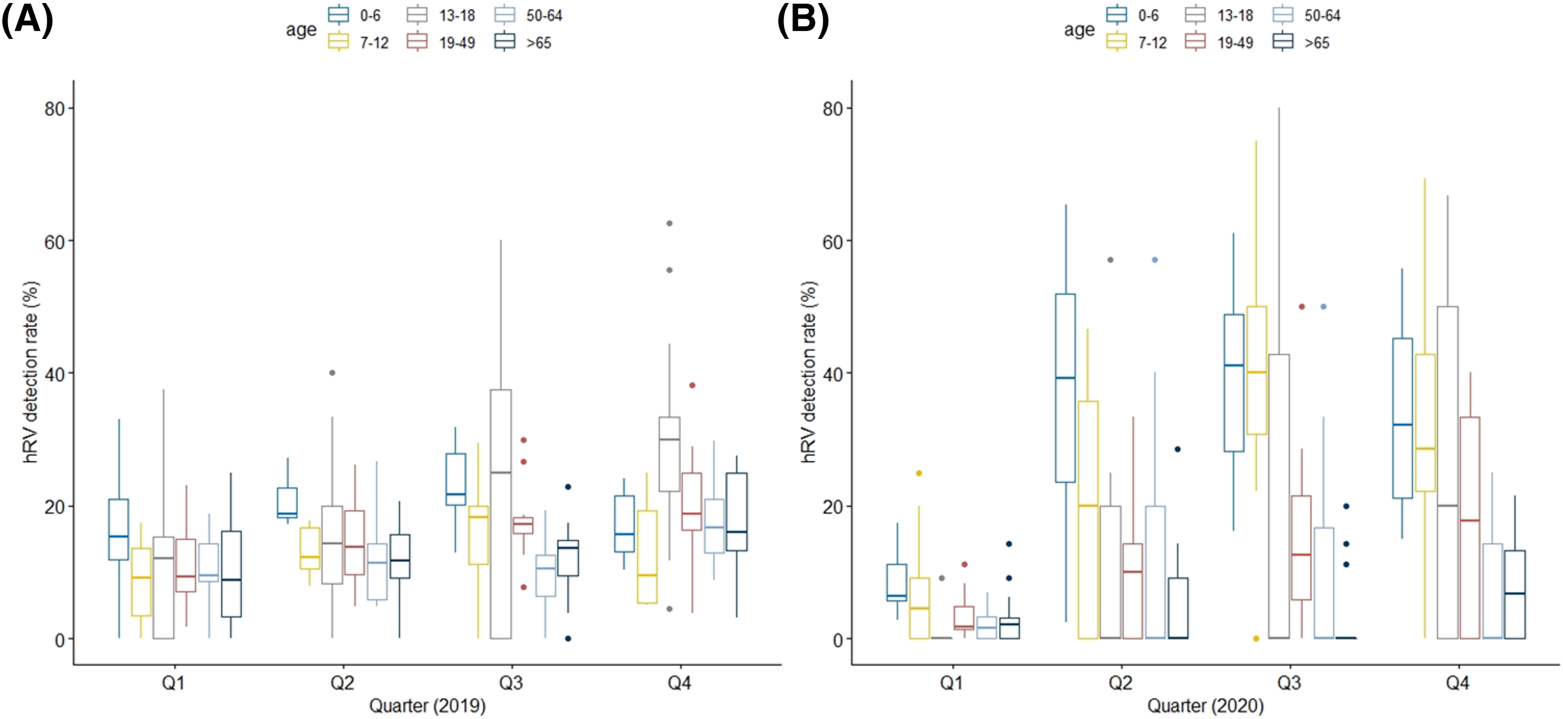
Comparison of human rhinovirus (hRV) detection rate between 2019 and 2020 on a quarterly basis by age. Age groups were divided into 0–6, 7–12, 13–18, 19–49, 50–64, and over 65 years

To compare hRV detection patterns by patient age and year, the detection rate of hRV in each quarter was compared among six age groups between 2019 and 2020. The lowest detection rate was observed in the first quarter of both 2019 and 2020. Although a low detection rate was maintained in the second quarter of 2019, the rate was increased in 2020, particularly in the 0–6 years group. In the third quarter, the mean detection rate of hRV in the 0–6 and 7–12 years groups was 40% in 2020 higher than that in 2019 but slightly decreased in the fourth quarter. In contrast, at 50–64 and over 65 years, the mean detection rate of hRV was around 10% in 2019, which was higher than that in 2020 (Figure 6).

### 3.4 Distribution of hRV species by age and year (2019–2020)

We investigated the distribution of hRV species over 2 years (2019–2020) in KINRESS. We randomly selected 52 and 42 respiratory specimens from 2019 and 2020, respectively, and the partial 5′ untranslated region and all of the VP4 and partial VP2 region (624 base pairs) were sequenced to identify the clinical strains (hRV A, B, and C). All sequences showed >98% similarity to hRV sequences with partial coding sequences available in GenBank. The A species was dominant in all age groups in both 2019 and 2020 at 76.9% and 85.7%, respectively. The hRV C species was detected in 15.4% and 11.9% of samples from 2019 and 2020, respectively, and frequently detected in the 0–6 and over 65 years groups. HRV B showed the lowest detection rates of 7.7% and 2.4% in 2019 and 2020, respectively, mostly in the 0–6 years group. There were no significant differences in the distribution of hRV species between 2019 and 2020 (Table 1).

**TABLE 1:** Number of hRV A, B, and C species detected by age in 2019 and 2020

Year	No. tested	No. detected				Total no. (%)	Number of hRV positive cases in each different species by age group																	
			No. (%) of hRV species detected				hRV A						hRV B						hRV C					
			A	B	C		0–6^a^	7–12^a^	13–18^a^	19–49^a^	50–64^a^	>65^a^	0–6^a^	7–12^a^	13–18^a^	19–49^a^	50–64^a^	>65^a^	0–6^a^	7–12^a^	13–18^a^	19–49^a^	50–64^a^	>65^a^
2019	13 010	2086	40 (76.9)	4 (7.7)	8 (15.4)	52 (100)	14	4	2	5	9	6	3	0	1	0	0	0	4	0	0	1	1	2
2020	6094	1067	36 (85.7)	1 (2.4)	5 (11.9)	42 (100)	18	2	8	6	1	1	1	0	0	0	0	0	3	0	0	1	0	1
Total	19 104	3153	76 (80.9)	5 (5.3)	13 (13.8)	94 (100)	32	6	10	11	10	7	4	0	1	0	0	0	7	0	0	2	1	3

*Note*: The age groups were divided as 0–6, 7–12, 13–18, 19–49, 50–64, and over 65 years.

Abbreviation: hRV, human rhinovirus.

^a^ The unit label is years old.

### 3.5 Comparison of genetic characteristics of hRV between 2019 and 2020

**FIGURE 7:**
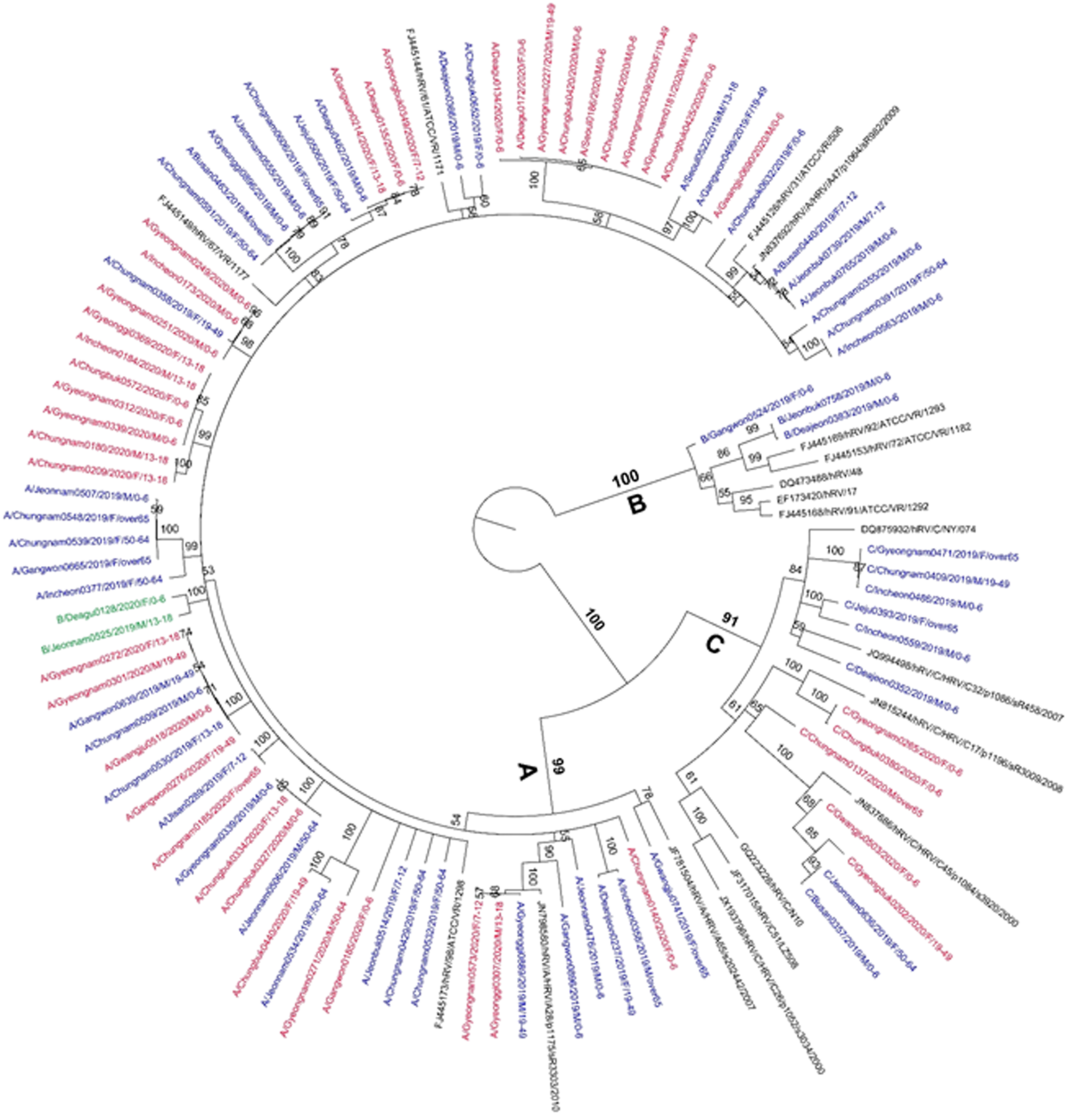
Maximum likelihood tree of the partial 5′ untranslated region/full VP4/partial VP2 regions, indicating human rhinovirus (hRV) species. The blue letters show the sequence of hRV detected in 2019, and the red letters indicate that in 2020. The black letters indicate the reference sequence of the prototypes. The nomenclature of hRV in the phylogenetic tree is as follows species (A, B, or C)/sentinel province and specimen no./detected year/gender of patient/age group

Figure 7 shows the maximum likelihood tree constructed using 52 sequences from 2019, 42 sequences form 2020, and 19 reference sequences^12^ from GenBank. Ninety‐four sequences were segregated into three phylogenetically distinct groups: 78 (83%) hRV A, 13 (13.8%) hRV C, and 3 (3.2%) hRV B. Two discordant hRV species were observed in the hRV A group in the unrooted maximum likelihood tree based on the NCBI BLAST results. Phylogenetic analysis indicated that most hRV species belonged to the hRV A group, showing mixed age, sex, region, and detection years without forming a separate cluster. Particularly, two amino acid deletions were observed at positions 64–65, corresponding to glycine‐isoleucine (i.e., LEK[GI]PTL in FJ445149 hRV ATCC VR‐1177), which were only detected hRV C in both 2019 and 2020. No genetic signature was identified in the hRV A, B, and C groups when hRV species were compared before COVID‐19 (2019) and after COVID‐19 (2020).

## 4 DISCUSSION AND CONCLUSION

COVID‐19 outbreak has not only caused public health challenges worldwide but also greatly impacted the economy, society, and normal daily lives.^19^, ^20^ Since COVID‐19 was first detected in January 20, 2020 in South Korea, it spread as a local epidemic among religious groups, schools, rallies, and even in family groups.^2^, ^3^, ^4^ The first wave of COVID‐19 occurred on March, 2020 leading to 200–300 patients becoming infected with severe acute respiratory syndrome coronavirus 2 in 1 day. After the first wave, intense social distancing and active mask‐wearing measures prevented the spread of COVID‐19 and led to reduced respiratory virus infections as well as changes in the detection patterns in KINRESS compared with that in previous years.^7^ The total number of specimens collected from the centers in 2020 was 6094, which is 53.2% lower than that in 2019. This may be because patients with ILI visited COVID‐19 screening centers rather than KINRESS centers. However, this did not significantly affect the analysis of the weekly detection rate of respiratory viruses. South Korea has four seasons (spring, summer, fall, and winter), and the detection patterns of respiratory viruses differ by season.^21^ However, during the COVID‐19 pandemic, enveloped viruses (IFV, hCoV, hPIV, hRSV, and hMPV) were rarely detected in patients with ILI in KINRESS. Only non‐enveloped viruses (hRV, hAdV, and hBoV) were detected, with the rate of hRV in 2020 higher than those in the previous 4 years (2016–2019). A high detection rate of hRV was also observed in private diagnostic sectors, which diagnose larger numbers of patients than the national surveillance (KINRESS). Although the number of patients with ILI was decreased because of social distancing and quarantine measures (such as wearing masks and washing hands properly), hRV showed a high detection rate, as it is a non‐enveloped virus that is resistant to environmental stress and exhibits prolonged viral shedding for an average of 10–14 days from immunocompetent subjects.^22^, ^23^

A negative correlation between influenza A virus and hRV has been observed in some studies as well as in KINRESS.^24^, ^25^ Since the start of the COVID‐19 pandemic, the detection rate of IFV has dramatically decreased, eventually reaching 0%. Rather than IFV, COVID‐19 showed a negative correlation with hRV. The hRV A species was detected in KINRESS in all age groups (0–6, 7–12, 13–18, 19–49, 50–64, and over 65 years) in 2019 and 2020. Interestingly, the hRV C species was detected mostly in the 0–6 years age group in 2019 and 2020, as observed previously.^26^, ^27^ It is thought that hRV C sensitivity is high in children with relatively low exposure to the virus.

The 94 sequences of the partial 5′ untranslated region and VP4‐partial VP2 coding region were segregated into three phylogenetically distinct groups: 78 (83.0%) hRV A, 13 (13.8%) hRV C, and 3 (3.2%) hRV B. Discordance was detected between two sequences (B/Deagu0128/2020/F/0–6 and B/Jeonnam0525/2019/M/13–18), which were confirmed as hRV B species by NCBI BLAST; however, they were grouped as hRV A species in the phylogenetic tree. Additional sequences for other capsids (VP3 and VP1) are required for the accordance in phylogenetic tree. However, we observed no differences in the genetic characteristics of hRV in 2020 compared with those in previous years based on the partial 5′ untranslated region and VP4‐partial VP2 coding regions. This result demonstrates that the high detection rate of hRV in 2020 when COVID‐19 was an epidemic did not occur because of changes in the genetic characteristics of hRV but rather because of the prolonged viral shedding period of hRV‐infected patients and its environmental resistance. Other study confirmed that clinical manifestations in patients with hRV C were more severe than in those with hRV A and B^28^, ^29^; however, the difference in clinical manifestations (fever, cough, sore throat, wheezing, chill, headache, muscle ache, nasal discharge, dyspnea, and phlegm) was not significant in this study (data not shown).

Social distancing and quarantine measures resulted in an unprecedented 0% influenza detection rate during national influenza surveillance in 2020, demonstrating that those are the most effective methods for preventing respiratory infectious diseases.^7^ Most countries are currently applying vaccination strategies to control COVID‐19, which are expected to change the course of the COVID‐19 epidemic by late 2021. Maintaining social distancing, quarantine measures and COVID‐19 vaccination programs will also alter the detection pattern of respiratory viruses. COVID‐19 is no longer an emerging infectious disease but must be subjected to surveillance as is performed for influenza. The World Health Organization recommended integrating COVID‐19 into the Global Influenza Surveillance and Response System.^30^, ^31^ Furthermore, it is crucial to monitor newly emerging infections by characterizing respiratory viruses other than COVID‐19.

## AUTHOR CONTRIBUTIONS

**Heui Man Kim:** Conceptualization; data curation; formal analysis; investigation; methodology; validation. **Eun Jung Lee:** Data curation; formal analysis; methodology; software. **Nam‐Joo Lee:** Investigation; methodology. **Sang Hee Woo:** Investigation; methodology. **Jeong‐Min Kim:** Investigation; methodology. **Jee Eun Rhee:** Conceptualization; validation. **Eun‐Jin Kim:** Supervision; validation.

## CONFLICT OF INTEREST

No conflict of interest declared.

## ETHICS APPROVAL

Ethical approval was obtained for the Korea Influenza and Respiratory Surveillance System, KINRESS from Ethics committee in Korea Disease Control and Prevention Agency, KDCA (2016‐05‐02‐C‐A). All necessary patients' consent has been obtained, and the appropriate institutional forms have been archived.

### PEER REVIEW

The peer review history for this article is available at https://publons.com/publon/10.1111/irv.12894.

## Supporting information


**Figure S1.** Comparison of detection rate of eight respiratory viruses in 2020 and recent 4 years (2016–2019). Welch's t‐test was applied for the statistical analysis.

**Table S1.** GeneBank accession numbers for part of the 5′ untranslated region and all of the VP4 and part of the VP2 regions of ninety‐four HRV used in this study.

## Data Availability

The weekly detection rate of influenza and other respiratory viruses in Korea Influenza and Respiratory Viruses Surveillance and System, KINRESS, is available on the Pathogens and Vector Surveillance Weekly Report in Korea Disease Control and Prevention Agency, KDCA website (http://www.kdca.go.kr/npt/biz/npp/portal/nppPblctDtaMain.do).

## References

[1] Zhu H , Wei L , Niu N . The novel coronavirus outbreak in Wuhan. China Glob Health Res Policy. 2020;5(1):6. 32226823 10.1186/s41256-020-00135-6 PMC7050114

[2] Kim JM , Chung Y‐S , Jo HJ , et al. Identification of coronavirus isolated from a patient in Korea with COVID‐19. Osong Public Health Res Perspect. 2020;11(1):3‐7. 32149036 10.24171/j.phrp.2020.11.1.02 PMC7045880

[3] Kim JH , An JA‐R , P‐k M , Bitton A , Gawande AA . How South Korea responded to the COVID‐19 outbreak in Daegu. NEJM Catal Innov Care Deliv. 2020;1.

[4] Seong H , Hyun HJ , Yun JG , et al. Comparison of the second and third waves of the COVID‐19 pandemic in South Korea: Importance of early public health intervention. Int J Infect Dis. 2021;104:742‐745. 33556610 10.1016/j.ijid.2021.02.004 PMC7863747

[5] Park SW , Sun K , Viboud C , Grenfell BT , Dushoff J . Potential role of social distancing in mitigating spread of coronavirus disease, South Korea. Emerg Infect Dis. 2020;26(11):2697‐2700. 32795385 10.3201/eid2611.201099 PMC7588540

[6] Park J , Chung E . Learning from past pandemic governance: Early response and Public‐Private Partnerships in testing of COVID‐19 in South Korea. World Dev. 2021;137:105198. 32982017 10.1016/j.worlddev.2020.105198 PMC7500944

[7] Kim HM , Lee H , Lee N‐j , Kim E‐J , et al. COVID‐19 impact on influenza and respiratory viruses surveillance. Public Health Weekly Report. 2020;13:3537‐3548.

[8] Choi WS . The national influenza surveillance system of Korea. Infect Chemother. 2019;51(2):98‐106. 31270989 10.3947/ic.2019.51.2.98 PMC6609753

[9] Fitzner J , Qasmieh S , Mounts AW , et al. Revision of clinical case definitions: Influenza‐like illness and severe acute respiratory infection. Bull World Health Organ. 2018;96(2):122‐128. 29403115 10.2471/BLT.17.194514 PMC5791775

[10] Ham H , Jang J , Jo S , Oh Y , Pak S . Infection frequency and mixed infection on eight viruses from patients with acute respiratory syndromes in Seoul. J Bacteriol Virol. 2014;44(3):274.

[11] R Core Team . R: A language and environment for statistical computing Vienna, Austria: R Foundation for Statistical Computing. 2020. https://www.R-project.org/

[12] Ratnamohan VM , Zeng F , Donovan L , MacIntyre CR , Kok J , Dwyer DE . Phylogenetic analysis of human rhinoviruses collected over four successive years in Sydney, Australia. Influenza Other Respi Viruses. 2016;10(6):493‐503. 10.1111/irv.12404 PMC5059946 27383422

[13] Jokela P , Joki‐Korpela P , Maaronen M , Glumoff V , Hyypiä T . Detection of human picornaviruses by multiplex reverse transcription‐PCR and liquid hybridization. J Clin Microbiol. 2005;43(3):1239‐1245. 15750090 10.1128/JCM.43.3.1239-1245.2005 PMC1081250

[14] Olive DM , Al‐Mufti S , Al‐Mulla W , et al. Detection and differentiation of picornaviruses in clinical samples following genomic amplification. J Gen Virol. 1990;71(9):2141‐2147. 2170576 10.1099/0022-1317-71-9-2141

[15] Benson DA , Cavanaugh M , Clark K , et al. GenBank. Nucleic Acids Res. 2012;41(D1):D36‐D42. 27899564 10.1093/nar/gkw1070 PMC5210553

[16] Kumar S , Stecher G , Tamura K . MEGA7: Molecular evolutionary genetics analysis version 7.0 for bigger datasets. Mol Biol Evol. 2016;33(7):1870‐1874. 27004904 10.1093/molbev/msw054 PMC8210823

[17] Silvestro D , Michalak I . raxmlGUI: A graphical front‐end for RAxML. Org Div Evol. 2012;12(4):335‐337.

[18] Darriba D , Taboada GL , Doallo R , Posada D . jModelTest 2: More models, new heuristics and parallel computing. Nat Methods. 2012;9(8):772. 10.1038/nmeth.2109 PMC4594756 22847109

[19] Ozili PK , Arun TG . Spillover of COVID‐19: Impact on the global economy. Available at SSRN 3562570 2020.

[20] Ammar A , Trabelsi K , Brach M , et al. Effects of home confinement on mental health and lifestyle behaviours during the COVID‐19 outbreak: Insights from the ECLB‐COVID19 multicentre study. Biol Sport. 2021;38(1):9‐21. 33795912 10.5114/biolsport.2020.96857 PMC7996377

[21] Kim J‐M , Jung H‐D , Cheong H‐M , et al. Nation‐wide surveillance of human acute respiratory virus infections between 2013 and 2015 in Korea. J Med Virol. 2018;90(7):1177‐1183. 29488229 10.1002/jmv.25069 PMC7166751

[22] To KKW , Yip CCY , Yuen K‐Y . Rhinovirus–from bench to bedside. J Formos Med Assoc. 2017;116(7):496‐504. 28495415 10.1016/j.jfma.2017.04.009

[23] Zlateva KT , de Vries JJC , Coenjaerts FEJ , et al. Prolonged shedding of rhinovirus and re‐infection in adults with respiratory tract illness. Eur Respir J. 2014;44(1):169‐177. 24876172 10.1183/09031936.00172113

[24] Nickbakhsh S , Mair C , Matthews L , et al. Virus–virus interactions impact the population dynamics of influenza and the common cold. Proc Natl Acad Sci U S A. 2019;116(52):27142‐27150. 10.1073/pnas.1911083116 PMC6936719 31843887

[25] Wu A , Mihaylova VT , Landry ML , Foxman EF . Interference between rhinovirus and influenza A virus: A clinical data analysis and experimental infection study. Lancet Microbe. 2020;1(6):e254‐e262. 33103132 10.1016/s2666-5247(20)30114-2 PMC7580833

[26] Cox DW , Khoo S‐K , Zhang G , et al. Rhinovirus is the most common virus and rhinovirus‐C is the most common species in paediatric intensive care respiratory admissions. Eur Respir J. 2018;52(2):1800207. 29976655 10.1183/13993003.00207-2018 PMC6295450

[27] Wildenbeest JG , van der Schee MP , Hashimoto S , et al. Prevalence of rhinoviruses in young children of an unselected birth cohort from the Netherlands. Clin Microbiol Infect. 2016;22:736.e9‐736.e15. 10.1016/j.cmi.2016.05.022 PMC7128250 27265373

[28] Piralla A , Rovida F , Campanini G , et al. Clinical severity and molecular typing of human rhinovirus C strains during a fall outbreak affecting hospitalized patients. J Clin Virol. 2009;45(4):311‐317. 19473873 10.1016/j.jcv.2009.04.016

[29] Bizzintino J , Lee W‐M , Laing IA , et al. Association between human rhinovirus C and severity of acute asthma in children. Eur Respir J. 2011;37(5):1037‐1042. 20693244 10.1183/09031936.00092410 PMC3024467

[30] World Health Organization . Public health surveillance for COVID‐19: Interim guidance, 16 December 2020. No. WHO/2019‐nCoV/SurveillanceGuidance/2020.8. World Health Organization, 2020.

[31] World Health Organization . Operational considerations for COVID‐19 surveillance using GISRS: Interim guidance, 26 March 2020. No. WHO/2019‐nCoV/Leveraging_GISRS/2020.1. World Health Organization, 2020.

