# Supplementary material for: Impact of coronavirus disease 2019 on respiratory surveillance and explanation of high detection rate of human rhinovirus during the pandemic in the Republic of Korea
Source: Influenza Other Respir Viruses. 2021 Aug 18;15(6):721–31. doi: 10.1111/irv.12894 (PMC8446939; doi:10.1111/irv.12894)
Supplement: Supplementary file 1 — Figure S1. Comparison of detection rate of eight respiratory viruses in 2020 and recent 4 years (2016–2019). Welch's t‐test was applied for the statistical analysis. [file IRV-15-721-s001.pptx]

## Slide 1
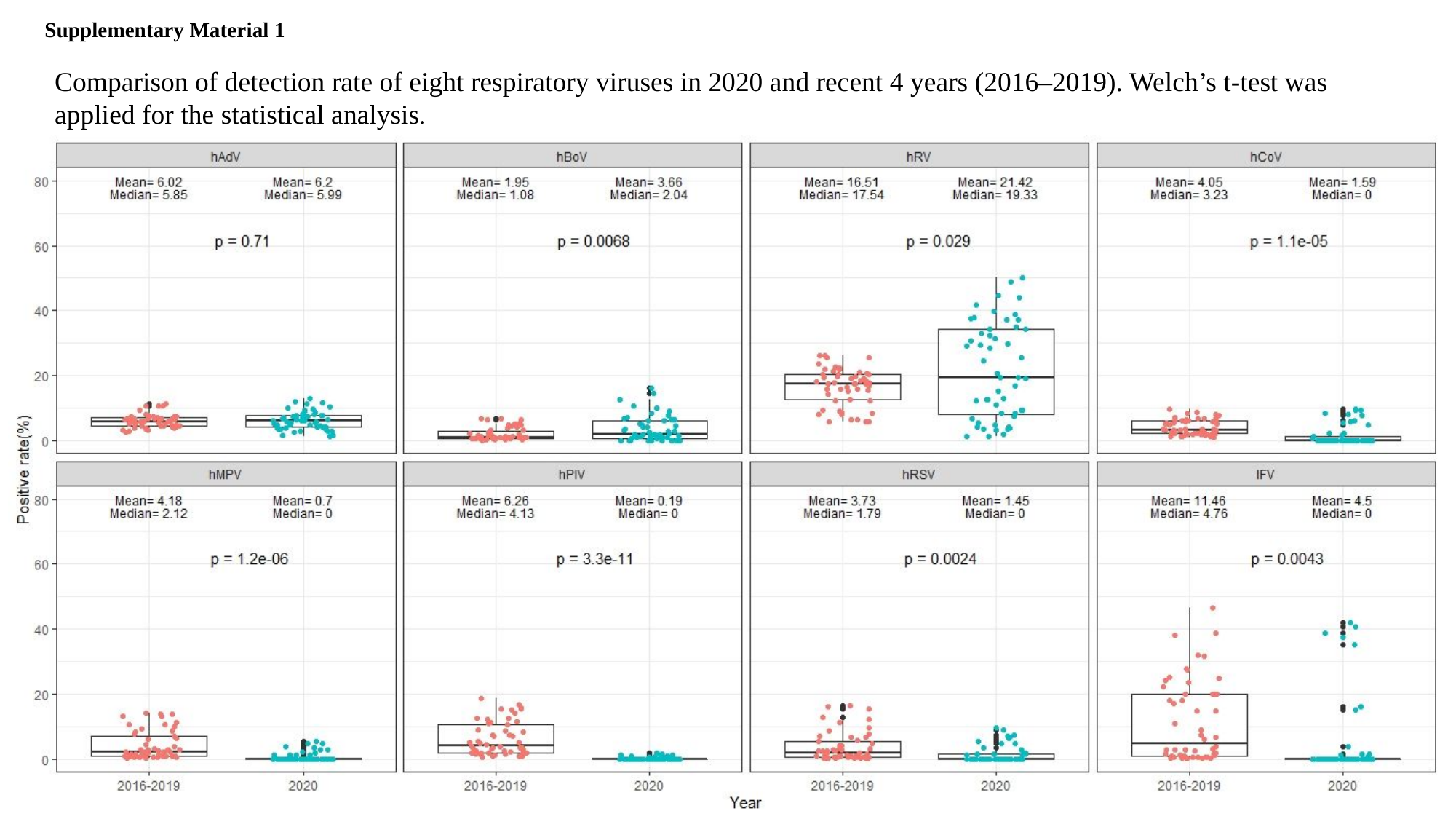

Supplementary Material 1
Comparison of detection rate of eight respiratory viruses in 2020 and recent 4 years (2016–2019). Welch’s t-test was applied for the statistical analysis.
